# Case report: Resolution of malignant canine mast cell tumor using ketogenic metabolic therapy alone

**DOI:** 10.3389/fnut.2023.1157517

**Published:** 2023-03-28

**Authors:** Thomas N. Seyfried, Purna Mukherjee, Derek C. Lee, Linh Ta, Loren Nations

**Affiliations:** ^1^Department of Biology, Boston College, Chestnut Hill, MA, United States; ^2^Veterinary Healthcare Associates, Winter Haven, FL, United States

**Keywords:** ketogenic diet, canine, glucose, aerobic fermentation, nutrition, calorie restriction

## Abstract

**Background:**

Mast cell tumors (MCT) are common neoplasms in dogs and are similar to most other malignant cancers in requiring glucose for growth, regardless of histological grade. Ketogenic metabolic therapy (KMT) is emerging as a non-toxic nutritional intervention for cancer management in animals and humans alike. We report the case of a 7 years-old Pit Bull terrier that presented in 2011 with a cutaneous mast cell tumor under the right nostril.

**Methods:**

The patient’s parent refused standard of care (SOC) and steroid medication after initial tumor diagnosis due to the unacceptable adverse effects of these treatments. Following tumor diagnosis, the patient’s diet was switched from Ol’Roy dog food to raw vegetables with cooked fish. The tumor continued to grow on this diet until July, 2013 when the diet was switched to a carbohydrate free, raw calorie restricted ketogenic diet consisting mostly of chicken and oils. A dog food calculator was used to reduce calories to 60% (40% calorie restriction) of that consumed on the original diet. A total of 444 kilocalories were given twice/day at 12 h intervals with one medium-sized raw radish given as a treat between each meal.

**Results:**

The tumor grew to about 3–4 cm and invaded surrounding tissues while the patient was on the raw vegetable, cooked fish diet. The tumor gradually disappeared over a period of several months when the patient was switched to the carbohydrate free calorie restricted ketogenic diet. The patient lost 2.5 kg during the course of the calorie restriction and maintained an attentive and active behavior. The patient passed away without pain on June 4, 2019 (age 15 years) from failure to thrive due to an enlarged heart with no evidence of mast cell tumor recurrence.

**Conclusion:**

This is the first report of a malignant cutaneous mast cell tumor in a dog treated with KMT alone. The resolution of the tumor in this canine patient could have been due to the diet-induced energy stress and the restriction of glucose-driven aerobic fermentation that is essential for the growth of most malignant tumors. Further studies are needed to determine if this non-toxic dietary therapeutic strategy could be effective in managing other canine patients with malignant mast cell tumors.

## Introduction

Canine cutaneous mast cell tumor (MCT) is a common malignant cancer in a range of dog breeds (1–4). Emerging evidence indicates that cancer is a mitochondrial metabolic disease (5, 6). Aerobic glucose fermentation (Warburg effect), linked to defective oxidative phosphorylation (OxPhos), has been documented in canine MCT as it has been in the majority of human cancers regardless of histological or genetic heterogeneity (7, 8). Unlike normal cells, cancer cells can grow in the absence of oxygen using glucose and glutamine as fermentable fuels (8). Ketogenic metabolic therapy (KMT) is a non-toxic therapeutic strategy for cancer management that restricts the availability of fermentable fuels while elevating levels of non-fermentable fatty acids and ketone bodies (9, 10). Metabolism of ketone bodies in normal cells increases the redox span between mitochondrial Complexes I and III, thus increasing the delta G’ of ATP hydrolysis while, at the same time, reducing the formation of reactive oxygen species (ROS) through the Complex II coenzyme Q couple (10–12). It is for these reasons that ketone bodies are considered *good medicine* for enhancing mitochondrial energy efficiency and general physiological health (13, 14). We therefore proposed that KMT might be helpful in managing canine MCT.

Calorie reduction and calorie restricted diets can also target multiple hallmarks of cancer including angiogenesis, inflammation, edema, and tumor cell viability (15–17). In contrast to cells with normal mitochondrial OxPhos capacity, cancer cells lack metabolic flexibility and cannot efficiently use fatty acids, ketone bodies, or other respiratory fuels for ATP synthesis (10). The well-documented abnormalities in mitochondrial number, structure, and function compromise energy synthesis through OxPhos (8). KMT used either alone or in combination with standard of care has therapeutic benefit for managing a broad range of animal and human cancers (9, 18–20). In this report, we present evidence showing that KMT used alone was able to resolve a malignant cutaneous MCT in a dog.

## Case report

A 7 years-old, 60 pound female Pit Bull (DOB, January 3, 2004) presented on July 28, 2011 at the Banfield Pet Hospital, Conyers, GA, USA with a cutaneous mass under the right nostril. Microscopic analysis of tissue smears revealed high cellularity with loose sheets of round to oval cells. The cells had indistinct borders and the cytoplasm contained large numbers of prominent metachromatic cytoplasmic granules. The nuclei varied mildly to moderately in size and nucleoli were rarely visible. Eosinophils were scattered frequently throughout the smear preparations. The cutaneous mass was diagnosed as mast cell tumor, but was not graded at that time (see lab report in Supplementary material). Based on clinical behavior, this tumor was likely a progressive low-grade neoplasm at the time of diagnosis (2–4). A complete blood work analysis revealed no deviations from normal ranges at the time of initial diagnosis (Supplementary material).

**FIGURE 1:**
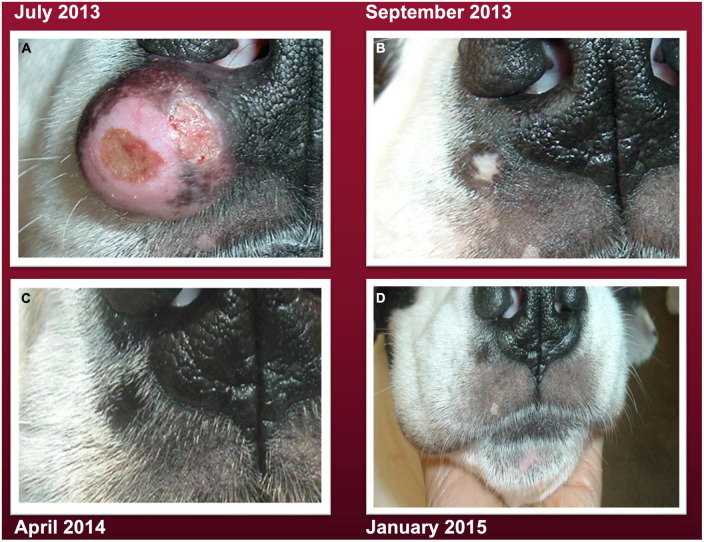
A large cutaneous mast cell tumors (MCT) (photographed on July 18, 2013) is seen under the right nostril and invasion to the nasal planum, consistent with malignancy (23). **(A)** The tumor gradually resolved over several months after the patient’s parent initiated the carbohydrate-free, calorie restricted ketogenic diet **(B–D)**, as described in methods. The small bare patch on the lip below the tumor can serve as a reference point to assess the degree of the diet-linked tumor shrinkage. A facial image from October 2016 is also shown in the Supplementary material.

Although steroid medication (prednisone) and standard of care (SOC) including surgery, chemotherapy, and radiation therapy were discussed as therapeutic options, the patient’s parent refused any of these treatments due to their unacceptable adverse effects. Significant adverse effects have been reported in a dog with cutaneous MCT treated with SOC (21, 22). Two weeks after tumor diagnosis, the patient’s diet was switched from Ol’Roy canned dog food to a raw vegetable diet with cooked fish and lentils. The tumor continued to grow on this diet invading local tissues and reaching a size of about 4 cm (Figure 1A). Local invasion is a hallmark of malignancy and the first step of the metastatic cascade (23, 24). Two weeks prior to the July 2013 image shown in Figure 1A, the patient’s diet was switched to a calorie restricted raw ketogenic diet. A full image of the patient’s face is shown in the Supplementary material. As no further diagnostic analysis was done on the tumor in 2013, it is not known if the grading of the tumor would have increased over the 2 years period from initial diagnosis in 2011 to that shown in Figure 1A and in the Supplementary material. The decision to switch the diet from raw vegetables to a raw calorie restricted KD was based on the on the view that dogs evolved from wolves, which are largely carnivores, and on the general information presented in the following YouTube video.^1^ Using the following dog food calculator, http://www.dogfoodadvisor.com/dog-feeding-tips/dog-food-calculator, the patient’s parent estimated that a 60 lb (27.3 kg), light-duty working dog should consume about 1,500 kilocalories (Kcal)/day. Consequently, a 40% restriction of this value was used to estimate a daily caloric intake for this patient of about 900 Kcal/day. The new diet was formulated to contain the following ingredients:

1. One organic raw chicken leg with bone (150 Kcal); 26 g fat: 36 g protein.
2. One organic raw chicken egg (54 Kcal); 1.5 g fat: 6.0 g protein.
3. One tablespoon (14.3 g) of pure LouAna coconut oil (120 calories); 14.3 g fat: 0 g protein.
4. Three teaspoons (12.6 g) of grizzly pollock oil for dogs (120 calories); 12.6 g fat: 0 g protein.

**FIGURE 2:**
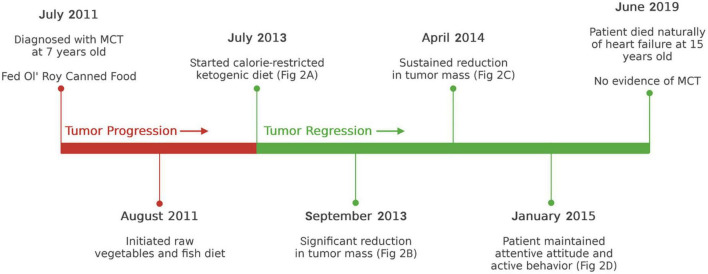
Timeline of clinical course with dates of dietary treatments.

This carbohydrate-free dietary formulation produced about 444 Kcal/meal with a fat: protein ketogenic ratio of about 1.3:1 and was fed to the patient twice/day in the morning and in the evening at 12 h intervals. There were no issues of compliance. As the patient enjoyed treats, one medium-sized raw radish (0 protein and 0 fat) was given between each meal. The tumor gradually disappeared over a period of several months when the patient was switched to the raw calorie restricted ketogenic diet (Figures 1B–D). A image of the patient’s face taken in 2016 is shown in the Supplementary material. The patient lost 2.5 kg (about 8% body weight) during the course of the calorie restriction and maintained an attentive and active behavior according to the parent. The patient was fed this calorie restricted ketogenic diet until 2019. Once the tumor was no longer apparent, the patient was occasionally given cooked chicken as an alternative to raw chicken. The patient passed away at the upper age of longevity for this breed (age 15 years) in the arms of the parent without pain on June 4, 2019 from failure to thrive due to an enlarged heart. No evidence of mast cell tumor recurrence was observed on the nose or anywhere else on the patient’s body. It is known that overall survival for grade II MCT is about 21.5 months and for grade III MCT is only about 9.2 months (2, 25). This patient survived for 63 months living a normal life span after resolution of the cancer. A timeline of the case is shown in Figure 2.

## Discussion

This is the first report to our knowledge of a malignant cutaneous MCT in a dog treated with KMT alone. It is clear from our observations that the KMT protocol used for our patient did not cause any adverse effects. No adverse effects or safety concerns were reported previously in canines treated with KD for managing epilepsy (26, 27). The diet-linked resolution of the tumor in this canine patient could have been due in part to the restriction of glucose and to an inhibition of aerobic fermentation (Warburg effect) that is essential for the growth of most malignant tumors (8, 28, 29). A limitation of our report, however, is the absence of data collected on blood glucose, blood ketone bodies, and glucose ketone index values (GKI) in a manner similar to those collected previously in our case report of a human brain tumor patient (30, 31).

It is recognized that the genome of most cancer cells, including those in MCT, contain numerous types of pathological somatic mutations (32–34). Abnormalities in mitochondrial DNA and biochemistry have also been reported previously in canine cutaneous MCT (7). These genetic abnormalities will prevent metabolic flexibility and adaptability to nutritional stress (35). Adaptability to abrupt environmental change is a property of the normal genome, which was selected for in order to ensure survival under environmental stress (35). According to established evolutionary concepts, only cells possessing flexibility in nutrient utilization will be able to survive under nutrient stress (6, 36). Environmental forcing over eons has selected for genomes that are capable of adapting to abrupt change in order to maintain metabolic homeostasis (37–39). Previous studies showed that normal dogs are remarkably adaptable to food restriction and physiological stress (40). The genomic defects that occur in MCT, together with mitochondrial dysfunction, will prevent the metabolic flexibility needed for rapid adaption to nutrient stress thus leading to tumor cell elimination through a combination of autophagy and autolytic cannibalism (36). The metabolically flexible normal canine cells will outcompete the mutated inflexible MCT cells for the availability of restricted nutrients thus leading to the elimination of the tumor cells (41, 42). It is therefore tempting to speculate that in contrast to the normal body cells, the neoplastic cells in the patient’s MCT were unable to adapt to the nutritional stress produced from the carbohydrate-free, calorie restricted ketogenic diet thus causing rapid tumor resolution.

Calorie restriction and restricted ketogenic diets have had success in reducing growth and metastasis in a range of malignant tumors in mice and humans (9, 19, 41, 43, 44). In none of these cases, however, was resolution of the tumor achieved with diet alone. Although a human glioblastoma patient has remained alive for over 8 years using KMT alone (no steroids, no radiation, no chemotherapy), the tumor in this patient was not resolved, but continues to grow slowly requiring periodic debulking for continued management (31). Synergy between restricted KD and glutamine targeting drugs could also facilitate resolution of more aggressive tumors especially for those that involve systemic metastasis and growth in the nervous system (16, 45). The resolution of the MCT in this canine patient should be viewed as anecdotal until further studies are conducted in other canine patients using a therapeutic strategy that is the same or similar to that used on our canine patient.

## Data availability statement

The raw data supporting the conclusions of this article will be made available by the authors, without undue reservation.

## Ethics statement

Ethical review and approval was not required for the animal study because there was no risk of toxicity or adverse events of the treatment recommended for the patient. Treatment was conducted in the patient’s home environment. Written informed consent was obtained from the owners for the participation of their animals in this study.

## Author contributions

TS wrote most of the report. PM, LT, and DL assisted with development of figures and report editing. LN edited and validated the accuracy of the conclusion. All authors contributed to the article and approved the submitted version.

## Footnotes

1
https://youtu.be/sBjnWfT8HbQ

## Supplementary material

1. Original lab report on the patient from the Banfield Pet Hospital, Conyers, GA.
2. Before and after ketogenic metabolic therapy (KMT) treatment images of the patient.

Supplementary Figure 1 Additional images depicting the patient before and after ketogenic metabolic therapy (KMT). **(A)** Large mast cell tumors (MCT) under the right nostril in July 2013. **(B)** Image of the patient with sustained resolution in October 2016.
