# Supplementary material for: Case report: Resolution of malignant canine mast cell tumor using ketogenic metabolic therapy alone
Source: Front Nutr. 2023 Mar 28;10:1157517. doi: 10.3389/fnut.2023.1157517 (PMC10086349; doi:10.3389/fnut.2023.1157517)
Supplement: Supplementary file 1 [file Data_Sheet_1.PDF]

Danial Moore
35 Village Walk
Covington, GA 30016
Home Phone: (770) 786-3891

Banfield Pet Hospital
1370 Dogwood Drive SE,
Conyers, GA 30013-5039
(770) 929-1222

Medical History Report

Laboratory Test Results

7/28/2011 - 7/28/2011

**Pet Information:**	**Species:** Canine	**Gender:** Female (Spayed)	**Birth Date:** 1/3/2004
**Name:** Nikaa**	**Breed:** Pit Bull	**Color:** White/Black	**Weight:** 60.00 Lbs/27.22 Kgs

Date/Time	Lab Test	Lab Result	Lab Unit	Evaluation	Lab Range	Results Date	Doctor	Hospital
7/28/2011 11:57:17 AM	Cytology					8/1/2011	Martin, V.	0279

Lab Comments: Microscopic Description: skin mass on nose
These smears are very cellular with single cells and loose sheets of monotypic round to oval cells. The cells vary mildly to moderately in cell and nuclear size, some have indistinct borders. Their cytoplasm has variably prominent metachromatic cytoplasmic granules, most cells are faintly, but well granulated. Nuclei are central and round, nucleoli are rarely visible. Eosinophils are scattered frequently throughout.

Microscopic Findings: Mast cell tumor

Comment: Cutaneous mast cell tumors in the dog have variable biologic behavior. This is partially dependent on grading, grading is evaluated by histopathology. Wide excision is advised, also check the draining lymph node for a metastatic lesion.

Cathy Thorn, DVM, DVSc, Diplomate A.C.V.P Veterinarians: Feel free to contact me if you have questions about this case at 800-872-1001, ext 3110

Date/Time	Lab Test	Lab Result	Lab Unit	Evaluation	Lab Range	Results Date	Doctor	Hospital
7/28/2011 11:42:24 AM	Tonometry - Left	20.0		Normal	(13.0-30.0)	7/28/2011	Martin, V.	0279
7/28/2011 11:42:24 AM	Tonometry - Right	20.0		Normal	(13.0-30.0)	7/28/2011	Martin, V.	0279
7/28/2011 11:42:24 AM	Albumin, Serum (ALB)	3.7	g/dL	Normal	(2.3-4.0)	7/28/2011	Martin, V.	0279
7/28/2011 11:42:24 AM	Alkaline Phosphatase (ALKP)	29.0	U/L	Normal	(23.0-212.0)	7/28/2011	Martin, V.	0279
7/28/2011 11:42:24 AM	ALT/SGPT (ALT)	31.0	U/L	Normal	(10.0-100.0)	7/28/2011	Martin, V.	0279
7/28/2011 11:42:24 AM	Amylase, Serum (AMYL)	605.0	U/L	Normal	(500.0-1,500.0)	7/28/2011	Martin, V.	0279
7/28/2011 11:42:24 AM	Calcium, Serum (CA)	10.3	mg/dL	Normal	(7.9-12.0)	7/28/2011	Martin, V.	0279
7/28/2011 11:42:24 AM	Cholesterol, Serum (CHOL)	262.0	mg/dL	Normal	(110.0-320.0)	7/28/2011	Martin, V.	0279
7/28/2011 11:42:24 AM	Creatinine, Serum (CREA)	1.4	mg/dL	Normal	(0.5-1.8)	7/28/2011	Martin, V.	0279

*** Indicates patient's invoice is still open

Danial Moore
35 Village Walk
Covington, GA 30016
Home Phone: (770) 786-3891

Banfield PET HOSPITAL

Banfield Pet Hospital
1370 Dogwood Drive SE,
Conyers, GA 30013-5039
(770) 929-1222

Medical History Report

Laboratory Test Results

7/28/2011 - 7/28/2011

**Pet Information:**	**Species:** Canine	**Gender:** Female (Spayed)	**Birth Date:** 1/3/2004
**Name:** Nikaa**	**Breed:** Pit Bull	**Color:** White/Black	**Weight:** 60.00 Lbs/27.22 Kgs

Date/Time	Lab Test	Lab Result	Lab Unit	Evaluation	Lab Range	Results Date	Doctor	Hospital
7/28/2011 11:42:24 AM	Glucose, Serum (GLU)	102.0	mg/dL	Normal	(74.0-143.0)	7/28/2011	Martin, V.	0279
7/28/2011 11:42:24 AM	Phosphorus, Serum (PHOS)	2.5	mg/dL	Normal	(2.5-6.8)	7/28/2011	Martin, V.	0279
7/28/2011 11:42:24 AM	Bilirubin, Total (TBIL)	0.4	mg/dL	Normal	(0.0-0.9)	7/28/2011	Martin, V.	0279
7/28/2011 11:42:24 AM	Protein, Total (TP)	7.0	g/dL	Normal	(5.2-8.2)	7/28/2011	Martin, V.	0279
7/28/2011 11:42:24 AM	BUN	19.0	mg/dL	Normal	(7.0-27.0)	7/28/2011	Martin, V.	0279
7/28/2011 11:42:24 AM	Globulin (GLOB)	3.2	g/dL	Normal	(2.5-4.5)	7/28/2011	Martin, V.	0279
7/28/2011 11:42:24 AM	Bands, Neutrophil, %	0.0	%	Normal	(0.0-2.0)	7/28/2011	Martin, V.	0279
7/28/2011 11:42:24 AM	Segs, Neutrophil, %	70.0	%	Normal	(60.0-77.0)	7/28/2011	Martin, V.	0279
7/28/2011 11:42:24 AM	Lymphocyte, %	15.0	%	Normal	(12.0-30.0)	7/28/2011	Martin, V.	0279
7/28/2011 11:42:24 AM	Eosinophil, %	10.0	%	Normal	(2.0-10.0)	7/28/2011	Martin, V.	0279
7/28/2011 11:42:24 AM	Basophil, %	0.0	%	Normal	(0.0-1.0)	7/28/2011	Martin, V.	0279
7/28/2011 11:42:24 AM	Monocyte, %	5.0	%	Normal	(3.0-10.0)	7/28/2011	Martin, V.	0279
7/28/2011 11:42:24 AM	Platelet Count (PLT)	439.0	10^3/µl	Normal	(200.0-500.0)	7/28/2011	Martin, V.	0279
7/28/2011 11:42:24 AM	RBC Morphology			Normal		7/28/2011	Martin, V.	0279
**Hospital Comments:** wnl								
7/28/2011 11:42:24 AM	Rouleau Formation - RBC			Negative		7/28/2011	Martin, V.	0279
7/28/2011 11:42:24 AM	Nucleated RBC			Negative		7/28/2011	Martin, V.	0279
7/28/2011 11:42:24 AM	WBC	5.18	10^3/µl	Below Normal	(6.0-17.0)	7/28/2011	Martin, V.	0279
7/28/2011 11:42:24 AM	RBC Count (RBC)	6.86	10^6/µl	Normal	(5.5-8.5)	7/28/2011	Martin, V.	0279
7/28/2011 11:42:24 AM	Hemoglobin (HGB)	16.43	g/dl	Normal	(12.0-18.0)	7/28/2011	Martin, V.	0279
7/28/2011 11:42:24 AM	Hematocrit (HCT)	47.86	%	Normal	(37.0-55.0)	7/28/2011	Martin, V.	0279
7/28/2011 11:42:24 AM	MCV	70.0	fl	Normal	(60.0-72.0)	7/28/2011	Martin, V.	0279
7/28/2011 11:42:24 AM	MCH	5.0	pg	Below Normal	(19.5-24.5)	7/28/2011	Martin, V.	0279

*** Indicates patient's invoice is still open

Danial Moore
35 Village Walk
Covington, GA 30016
Home Phone: (770) 786-3891

Banfield PET HOSPITAL

Banfield Pet Hospital
1370 Dogwood Drive SE,
Conyers, GA 30013-5039
(770) 929-1222

Medical History Report

Laboratory Test Results

7/28/2011 - 7/28/2011

**Pet Information:**	**Species:** Canine	**Gender:** Female (Spayed)	**Birth Date:** 1/3/2004
**Name:** Nikaa**	**Breed:** Pit Bull	**Color:** White/Black	**Weight:** 60.00 Lbs/27.22 Kgs

Date/Time	Lab Test	Lab Result	Lab Unit	Evaluation	Lab Range	Results Date	Doctor	Hospital
7/28/2011 11:42:24 AM	MCHC	34.33	g/dl	Normal	(34.0-38.0)	7/28/2011	Martin, V.	0279
7/28/2011 11:42:24 AM	RDW	14.4	%	Normal	(12.0-16.0)	7/28/2011	Martin, V.	0279
7/28/2011 11:42:24 AM	Platelet Count (PLT)	439.0	10^3/µl	Normal	(200.0-500.0)	7/28/2011	Martin, V.	0279
7/28/2011 11:42:24 AM	MPV	7.63	fl	Normal	(6.1-10.1)	7/28/2011	Martin, V.	0279
7/28/2011 11:42:24 AM	Fecal - Roundworms Eggs Observed			Negative		7/28/2011	Martin, V.	0279
7/28/2011 11:42:24 AM	Fecal - Hookworms Eggs Observed			Negative		7/28/2011	Martin, V.	0279
7/28/2011 11:42:24 AM	Fecal - Whipworm Eggs Observed			Negative		7/28/2011	Martin, V.	0279
7/28/2011 11:42:24 AM	Fecal - Giardia Observed			Negative		7/28/2011	Martin, V.	0279
7/28/2011 11:42:24 AM	Fecal - Coccidia Oocysts Observed			Negative		7/28/2011	Martin, V.	0279
7/28/2011 11:42:24 AM	Fecal - Tapeworms Observed			Negative		7/28/2011	Martin, V.	0279
7/28/2011 11:42:24 AM	Fecal - Abnormal Bacteria Observed			Negative		7/28/2011	Martin, V.	0279
7/28/2011 11:42:24 AM	Fecal - Significant Blood Observed			Negative		7/28/2011	Martin, V.	0279
7/28/2011 11:42:24 AM	Fecal - 'Other' Eggs Observed			Negative		7/28/2011	Martin, V.	0279

*** Indicates patient's invoice is still open
