# Supplementary figures and images for: Case report: Resolution of malignant canine mast cell tumor using ketogenic metabolic therapy alone
Source: Front Nutr. 2023 Mar 28;10:1157517. doi: 10.3389/fnut.2023.1157517 (PMC10086349; doi:10.3389/fnut.2023.1157517)

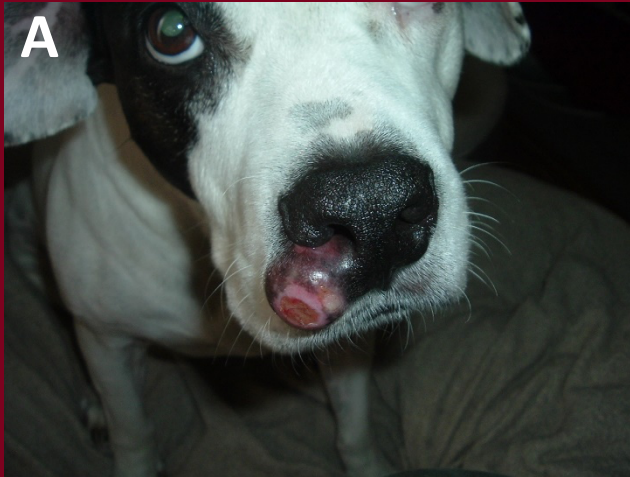

**July 2013**

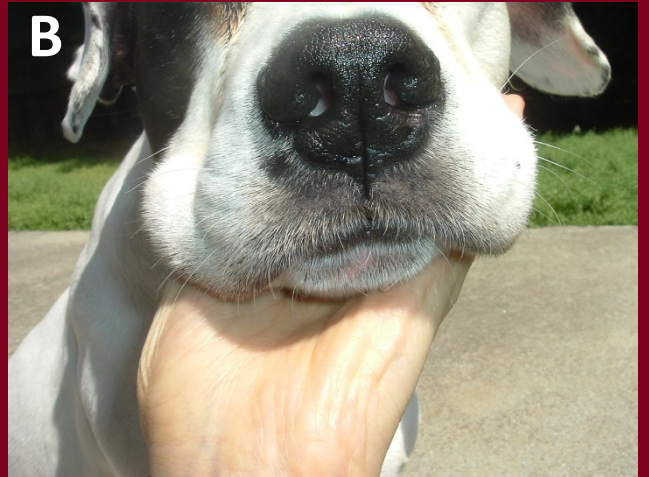

**October 2016**

Supplement: Supplementary Figure 1 — Additional images depicting the patient before and after ketogenic metabolic therapy (KMT). (A) Large mast cell tumors (MCT) under the right nostril in July 2013. (B) Image of the patient with sustained resolution in October 2016. [file Data_Sheet_2.PDF]
